# Medical Opinion and Movement

**Published:** 1907-03-23

**Authors:** 


### Medical Opinion and Movement.

In recent years there has been an overwhelming tendency for abdominal conditions of all kinds to pass from the domain of the physician to that of the surgeon. Improved methods of technique have so reduced the mortality rate that the surgeon has come to regard the risks of laparotomy *per se* as almost negligible. The extreme sensitiveness of infants, however, to any surgical manipulations of their internal organs has so far formed a serious obstacle to the forward march of the surgeon in infantile diseases. Operative interference in these little patients is so frequently and unexpectedly attended with a fatal result, for which no cause can be assigned apart from a hypothetical condition of shock. The surgeon is therefore naturally somewhat chary of incurring responsibility in these cases, in which he cannot accurately take measure of all the factors which will ensure success. It is not surprising to find in view of these facts that at the discussion on the treatment of congenital pyloric stenosis at the last meeting of the Clinical Society there was a considerable consensus of opinion among surgeons as well as physicians in favour of the medical treatment of this condition by careful feeding and by lavage of the stomach.

Papers were contributed by Dr. G. A. Sutherland, Dr. A. F. Voelcker, and Mr. F. F. Burghard. Dr. Sutherland's paper was devoted to a consideration of the medical treatment of the disease, illustrated by cases under his care. He dwelt on the importance of diet. Breast-feeding is the best; otherwise modified cow's milk, preferably peptonised, whey and Allenbury's food (No. 1) are in his experience the most suitable substitutes. Small feeds should be given frequently. In bad cases it may be necessary even to feed every hour by day and every two hours by night. Fatty constituents should be kept low, as they are especially liable to increase gastric irritation and vomiting. Gastric lavage should be carried out for some time daily, and in bad cases it may be desirable to perform it twice a day. Not only does lavage alleviate the condition, but it enables the physicians to judge by the residues how the food is being digested and to modify the diet accordingly. Feeding may be supplemented by normal saline injections per rectum until the pyloric function has so far improved as to allow of the absorption of sufficient fluid into the system by the natural route. Dr. Sutherland does not find sedative drugs of any value in relieving the pyloric spasm. In discussing operative treatment it was generally agreed that dilatation of the pylorus offered the best chances of success.

One of the most recent advances in surgical rhinology is the procedure of intranasal suturing, as carried out by Mr. Sidney Yankaner, of New York. In operations for the removal of a hypertrophied turbinate, for instance, he does not leave the wound to heal by granulations, but closes it by sutures, and in this way secures much more rapid healing by primary union. He has devised special instruments for this purpose, which no doubt require some manipulative skill in handling. The method is as follows: By means of a needle shaped like a Deschamps ligature-carrier (right or left as needed, and sharp at the point) a catgut stitch is passed through both edges of the wound in the mucous membrane. The suture end is then caught with a small hook and pulled out of the nose, and the suture-carrier is withdrawn. A single knot is tied in the catgut, one end of which is passed through the eye of an instrument consisting of a slender shank bearing a ring at its extremity. With this, acting practically as a prolongation of the index finger, the knot is pushed up into the nose. A second knot is similarly carried up to the wound, and the suture ends are cut off. The remaining stitches are introduced in a like manner. Not only is more rapid healing promoted, but the chances of secondary hæmorrhage or inflammatory infiltration are greatly reduced. Furthermore, by this method of suturing Mr. Yankaner has succeeded in closing perforations of the septum.

Our legislators, like the rest of the community, exhibit an extraordinary apathy in regard to questions of hygiene. This undoubtedly arises entirely from ignorance. If any sort of understanding could be instilled into the mind of the public of the importance of hygiene in relation to their well-being and efficiency a more serious interest might be displayed in measures to promote it. A more proper care of the health, individually and collectively, might undoubtedly be secured by inculcating the elementary principles of hygiene in the schools together with the three R's. In the course of the debate in Committee of Supply, Dr. Rutherford appealed to the President of the Board of Education to make the teaching of hygiene and temperance compulsory in all elementary schools. He pointed out that two years ago Parliament had been petitioned on the subject by a very large number of medical men, and it was much to be regretted that steps had not yet been taken to carry out this recommendation. He suggested further that the Board of Education should have a medical bureau to guide and help in this and other subjects. Mr. McKenna, in reply, promised his earnest attention to the matter, but pointed out that competent teachers were necessary, if these subjects were made compulsory, and that everything was being done to qualify teachers to give instruction in these subjects. It is difficult to understand why the necessity for competent teachers should be anything but a temporary obstacle to the inclusion of hygiene as a compulsory subject in the education code. The Board has but to take the necessary steps to make the subject compulsory within a certain period of time, and there is little doubt that teachers will quickly avail themselves of any facilities offered them to make themselves competent to teach it.

